# A Characterization of Biological Activities and Bioactive Phenolics from the Non-Volatile Fraction of the Edible and Medicinal Halophyte Sea Fennel (*Crithmum maritimum* L.)

**DOI:** 10.3390/foods13091294

**Published:** 2024-04-23

**Authors:** Clément Lemoine, Maria João Rodrigues, Xavier Dauvergne, Stéphane Cérantola, Luísa Custódio, Christian Magné

**Affiliations:** 1 Laboratoire Géoarchitecture_Territoires, Urbanisation, Biodiversité, Environnement, Université de Brest, CS 93837, F 29238 Brest, Cedex 3, France; lemoine1clement@gmail.com (C.L.); xavier.dauvergne@univ-brest.fr (X.D.); 2 Centre of Marine Sciences, Faculty of Sciences and Technology, University of Algarve, Ed. 7, Campus of Gambelas, 8005-139 Faro, Portugal; mjrodrigues@ualg.pt (M.J.R.); lcustodio@ualg.pt (L.C.); 3 Service Général des Plateformes Technologiques, Plateforme RMN-RPE, Université de Bretagne Occidentale, 6 av. le Gorgeu, CS 93837, F 29238 Brest, Cedex 3, France; stephane.cerantola@univ-brest.fr

**Keywords:** *Crithmum maritimum*, halophyte, antioxidants, anti-inflammatory agents, anti-ageing activity, antidiabetic activity, bioguiding, NMR characterization, phenolic compounds

## Abstract

Although the biochemical composition and biological properties of the volatile fraction of the halophyte sea fennel (*Crithmum maritimum* L.) have been largely described, little is known about its polar constituents and bioactivities. Here, a hydromethanolic extract of *Crithmum maritimum* (L.) leaves was fractionated, and the fractions were evaluated in vitro for antioxidant (using DPPH, ABTS, and FRAP bioassays), anti-inflammatory (inhibition of NO production in RAW 264.7 macrophages), antidiabetic (alpha-glucosidase inhibition), neuroprotective (inhibition of acetylcholinesterase), and skin-protective (tyrosinase and melanogenesis inhibitions) activities. Polar fractions of the extract were rich in phenolics and, correlatively, displayed a strong antioxidant power. Moreover, fractions eluted with MeOH_20_ and MeOH_80_ exhibited a marked inhibition of alpha-glucosidase (IC_50_ = 0.02 and 0.04 mg/mL, respectively), MeOH_60_ fractions showed a strong capacity to reduce NO production in macrophages (IC_50_ = 6.4 μg/mL), and MeOH_80_ and MeOH_100_ fractions had strong anti-tyrosinase activities (630 mgKAE/gDW). NMR analyses revealed the predominance of chlorogenic acid in MeOH_20_ fractions, 3,5-dicaffeoylquinic acid in MeOH_40_ fractions, and 3-*O*-rutinoside, 3-*O*-glucoside, 3-*O*-galactoside, and 3-*O*-robinobioside derivatives of quercetin in MeOH_60_ fractions. These compounds likely account for the strong antidiabetic, antioxidant, and anti-inflammatory properties of sea-fennel polar extract, respectively. Overall, our results make sea fennel a valuable source of medicinal or nutraceutical agents to prevent diabetes, inflammation processes, and oxidative damage.

## 1. Introduction

Plants have served as a valuable source of medicinal compounds for centuries, with plant-derived products being extensively used in various industries such as food, nutraceuticals, and medicine. The current shift towards natural remedies has spurred the exploration of bioactive components sourced from plants. For example, the antioxidant properties of plants have garnered significant interest in recent years, with the supplementation of exogenous antioxidants emerging as a promising strategy to combat the detrimental effects of oxidative stress [1,2].

Halophytes, in this context, offer a sustainable reservoir of bioactive substances that can be harnessed for applications in food, cosmetics, and pharmaceuticals. Despite being relatively understudied until the past decade, recent research has unveiled the rich secondary metabolite content of these plants, which underpins their diverse biological effects [3,4,5]. Among these compounds, polyphenols stand out for their potent antioxidant capabilities and broad spectrum of medicinal benefits [6,7,8,9].

**Figure 1:**
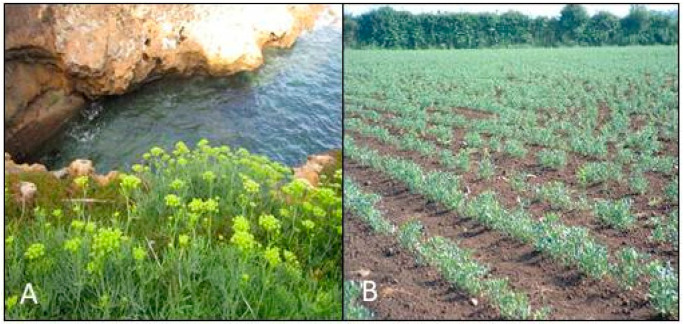
Plants of sea fennel on a maritime rocks (**A**) and grown in open field (**B**). (Source: C. Magné).

*Crithmum maritimum* L. (Apiaceae), also known as sea fennel or rock samphire, is a widespread perennial halophyte which grows mostly on coastal rocks, and sometimes on sandy beaches or pebbles, along the shoreline of the Mediterranean Sea and of the Pacific and Atlantic oceans. Alternatively, it can be grown easily in open air under agronomical techniques (Figure 1), producing 1.22 TDW/ha^−1^ (C. Magné, unpublished results). This facultative halophyte is edible and has a history of being utilized in culinary dishes and traditional remedies. Its juicy leaves and fresh shoots are preserved in vinegar for flavoring purposes. Moreover, *C. maritimum* contains high amounts of minerals, vitamins (A, C, E), carotenoids, flavonoids [10,11], and essential oils [12]. These confer to the plant antiscorbutic, carminative, cytotoxic, digestive, depurative, diuretic, tonic, vermifuge, and insecticidal properties [12,13]. Although the components present in the essential oils of *C. maritimum* and their properties have been largely studied [14,15,16,17] and have found many applications, little is known about the nonvolatile fraction in this species [18]. In a recent study, mineral and phenolic compositions in sea fennel were reported, but the extraction process used (leaf decoction) was not exhaustive for phenolic compounds [19]. Lastly, the NMR characterization of phenolic compounds from *C. maritimum* leaves, and their antimicrobial and antiproliferative activities, were reported [20]. However, the work carried out on raw extracts did not allow for the characterization of flavonoid compounds. Therefore, a bioguiding study reporting the characterization of sea-fennel phenolics was exhaustively extracted from the leaves, and, relating it to some biological activities of medicinal interest, is still lacking.

We have previously identified free quinic acid, a carbohydrate-derived secondary metabolite previously reported in other members of Apiaceae, and chlorogenic acid as major solutes in *C. maritimum*, with possible consequences on plant extract antioxidant activity [21]. Here, a hydromethanolic extract of *C. maritimum* leaves was fractionated to characterize for the first time anti-inflammatory, neuroprotective, and anti-ageing activities, as well as the relevant bioactive compounds.

## 2. Materials and Methods

### 2.1. Chemicals, Culture Media and Supplements

Acetylcholinesterase (EC 3.1.1.7), α-amylase (EC 3.2.1.1), α-glucosidase (EC 3.2.1.20), lipase (EC 3.1.1.3), and tyrosinase (EC 1.14.18.1) enzymes, as well as murine RAW 264.7 macrophages and B16 A45 melanoma cell lines, were purchased from Sigma-Aldrich (Darmstadt, Germany). Additionally, all reagents (ATChI, ferric chloride, DTNB, Folin–Ciocalteu phenol, L-glutamine, penicillin, potassium persulfate, streptomycin, and TPTZ), standards (acarbose, galantamine, gallic and kojic acids, L-NAME, melanine, and orlistat), radicals (DPPH and ABTS), and solvents (DMSO and methanol and ethanol of analytical grade) used for chemical analyses were supplied by Sigma-Aldrich (St. Louis, MO, USA).

### 2.2. Plant Material and Sampling

*C. maritimum* plants were collected on maritime rocks along the Brittany shore, near Brest (48°21′47″ N 4°31′51″ O, France). The plant material was identified by C. Magné (Faculty of Sciences, Brest). A voucher specimen was deposited at the herbarium of Sciences and Techniques Faculty, University of Brest. The aerial parts were rinsed and the leaves were placed at −20 °C for 5 days before being subjected to freeze-drying. Dried leaves (a_w_ < 0.3) were then pulverized into a fine powder with a Dangoumeau-type grinder and passed through a fine-mesh sieve to obtain a powder with a diameter <1 mm. The dry powder was finally stored at −20 °C without light, before being used for extraction and analyses.

### 2.3. Extraction and Fractionation

About 200 mg of powder underwent homogenization with 5 mL of water/ethanol (1:2) under magnetic stirring at 4 °C for 20 min. Following the centrifugation of the mixture (15 min at 4 °C, 4000× *g*), the resulting pellet was subjected to extraction twice using the same procedure. The supernatants were combined, filtered through glass wool, and concentrated through rotary evaporation at 40 °C, using 50% ethanol for resuspension.

To fractionate the initial extract, solid–liquid partition chromatography was conducted on C18-bound silica gel (GRACE Davisil RP18). Solutes with decreasing polarity were eluted using methanol in increasing concentrations (successively, 0, 20, 40, 60, 80, and 100%), ultimately followed by ethyl acetate. The collected fractions were then concentrated through rotary evaporation at 40 °C and resuspended in their respective solvents prior to analysis. Then, all analyses were carried out in triplicate for three independent samples.

### 2.4. Estimation of Total Phenolic Content (TPC)

The TPC of the extract and fractions was assayed according to the method described by Zhang et al. [22]. Twenty μL of sample solution was mixed with 100 μL Folin–Ciocalteu reagent in a 96-well microplate. After 5 min, 80 μL of 7.5% sodium carbonate (*w*/*v* in water) was added. The microplate was gently shaken, allowed to stand at room temperature for 2 h, and its absorbance was read at 750 nm with a microplate reader (Multiskan FC, Thermo Scientific Technologies^®^, Waltham, MA, USA). The TPC of the samples was expressed as gallic acid equivalents (GAEs) per gram of dry weight from a calibration curve of gallic acid (0–500 mg/L).

### 2.5. Antioxidant Activities

#### 2.5.1. DPPH Scavenging Activity

The scavenging activity of the stable 1,1-diphenyl-2-picrylhydrazyl (DPPH) free radical was determined by the method of Marwah et al. [23]. Briefly, the reaction medium contained 100 μL of 100 μM DPPH violet solution in ethanol and 100 μL of plant extract at different concentrations (or water for the control). The reaction mixture was incubated in the dark for 15 min and the absorbance was recorded at 517 nm. The assay was carried out in triplicate. The decrease in absorbance upon the addition of test samples was used to calculate the inhibition percentage (%IP) of DPPH radicals, following the following equation:

%IP = [(A_c_ − A_s_)/A_c_] × 100

where A_c_ and A_s_ are the absorbances of the control and the test sample, respectively. From a plot of concentration against %IP, a linear-regression analysis was performed to determine the antiradical activity, as expressed by the IC_50_ (extract concentration resulting in a 50% inhibition) value for each sample.

#### 2.5.2. Ferric Reducing Activity (FRAP)

The assay is based on the reaction of Fe^2+^ with 2,4,6-tri(pyridyl)-*s*-triazine (TPTZ) to form a violet-blue color with maximal absorbance at 593 nm [24]. The FRAP solution was prepared by mixing 10 volumes of acetate buffer (300 mM, pH 3.6) with 1 volume of TPTZ (40 mM in HCl) and 1 volume of ferric chloride (20 mM in water). The solution was prepared daily and warmed at 37 °C for 10 min before use. A 280 μL aliquot of this solution was mixed with 20 μL of samples (extract, fractions, or water for the blank) in a 96-well microplate. The mixture was incubated at 37 °C in the dark for 30 min and then read at 593 nm with a Multiskan FC microplate reader (Thermo Scientific Technologies, Shanghai, China). The increase in absorbance upon the addition of test samples was used to calculate the reducing capacity, as expressed by the following efficacy percentage (%EP):

%EP = [(A_s_ − A_c_)/A_c_] × 100

where A_s_ and A_c_ are the absorbances of the control and the test sample, respectively. From a plot of concentration against %EP, a linear-regression analysis was performed to determine the EC_50_ (extract concentration resulting in a 50% efficacy) value for each sample.

#### 2.5.3. ABTS Scavenging Activity

The ABTS radical scavenging assay was based on the method described by Re et al. [25] with a slight modification. Briefly, a 7 mM ABTS stock solution was prepared by dissolving ABTS in ethanol–water (5:1 *v*/*v*). Then, an aliquot of this solution was reacted with 2.45 mM potassium persulfate in ethanol–water (1:3 *v*/*v*) and allowed to stand in the dark at room temperature for 16–20 h to prepare the ABTS radical cation (ABTS^+^). This ABTS radical solution was diluted to an absorbance at 734 nm of 0.70 ± 0.02. Finally, the absorbance of a mixture consisting of the sample (or water for the blank) and the ABTS reagent was followed at 734 nm. The antiradical capacity of the samples was expressed as gallic acid equivalents.

### 2.6. Anti-Ageing Activity

An anti-tyrosinase assay was performed using L-DOPA as a substrate, according to the method described by Masuda et al. [26]. The samples (plant extract or fractions) were dissolved in 50% DMSO. Then, 40 μL of each sample was mixed with 80 μL of phosphate buffer (0.1 M, pH 6.8), 40 μL of tyrosinase (31 units/mL in phosphate buffer, pH 6.5), and 40 μL of 2.5 mM L-DOPA in a 96-well microplate. The absorbance of the mixture was measured at 475 nm and compared to those of a positive control containing kojic acid and a blank containing all the components except L-DOPA. The anti-tyrosinase activity was expressed as kojic acid equivalents (mg KAE/g DW).

Moreover, the anti-melanogenic activity of sea-fennel extract and fractions was assessed in vitro on B16 4A5 melanoma cells according to Bouzaiene et al. [27]. The cells were seeded at 3.5 × 10^4^ cells/well into 12-well plates, allowed to adhere for 24 h, and then treated with sea-fennel-extract concentrations that allowed for cellular viability higher than 80% for 72 h. Thereafter, adherent cells were trypsinized and solubilized in 1 mL of sodium dodecyl sulphate (SDS; 1%, *v*/*v*). The absorbance of the samples was measured at 475 nm, and the melanin content was estimated using a standard curve of synthetic melanin (0–25 μg/mL).

### 2.7. Neuroprotective Activity

The neuroprotective property of sea-fennel extract was evaluated through the in vitro inhibition of acetylcholinesterase (AChE) according to Custódio et al. [28]. Samples (20 µL at concentrations of 1, 5, and 10 mg/mL) were mixed with 140 µL of sodium phosphate buffer (0.1 mM, pH 8.0) and 20 µL of AChE solution (0.28 U/mL) in a 96-well microplate. The mixture was incubated for 15 min at room temperature and the reaction was initiated by the addition of 10 µL of 4 mg/mL ATChI and 20 µL of 1.2 mg/mL DTNB. The absorbance was read at 405 nm and results were expressed as IC_50_ relative to a control containing water instead of extract. Galantamine was used as a positive control.

### 2.8. Antidiabetic Activity

Sea-fennel extract and fractions, at concentrations ranging from 1 to 5 mg/mL, were evaluated for their capacity to inhibit α-amylase and α-glucosidase according to Zengin [29]. Moreover, extracts and fractions were tested against porcine lipase, according to McDougall et al. [30]. Acarbose was used as a positive control for α-amylase and α-glucosidase, and orlistat was used as a positive control for lipase inhibition. The results are expressed as IC_50_ relative to a control containing DMSO.

### 2.9. Anti-Inflammatory Activity

Nitric oxide (NO) production by LPS-stimulated RAW 264.7 macrophages was assessed as described by Rodrigues et al. [4]. RAW 264.7 cells were cultured in RPMI 1640 culture medium, enriched with 10% heat-inactivated FBS, 1% L-glutamine (2 mM), and 1% penicillin (50 U/mL)/streptomycin (50 μg/mL), and kept at 37 °C in a 5% CO_2_ humidified atmosphere. Murine cells were seeded in a 96-well plate at 2.5 × 10^5^ cells/well and allowed to adhere overnight. Then, they were co-treated with 100 ng/mL of LPS and sea-fennel raw extract (at concentrations that allowed for cellular viability higher than 80%) for 24 h. NO production was assessed using the Griess assay. The results are expressed as a percentage of inhibition of NO production, relative to a control containing DMSO (0.5%, *v*/*v*), and compared to the positive control L-NAME.

### 2.10. Solute Purification

When required, the purification of solutes from a specific fraction was carried out by HPLC using the Shimadzu UFLC XR device equipped with a PDA detector (SPD-M20A, Shimadzu, Kyoto, Japan). For solute separation, a Spherisorb ODS2 column (5 µm, 250 × 4.6 mm, Waters, Milford, MA, USA) was used, and the mobile phase consisted of a mixture of 100% acetonitrile (A) and ultrapure water (B). The purification process involved the following linear gradient: t = 0 min 100% B; t = 10 min 100% A. The compounds were detected at 254 nm and collected for acid hydrolysis treatment (1 N HCl, 110 °C for 1 h) prior to structural elucidation.

### 2.11. NMR Analyses

To characterize bioactive compounds, a portion of crude extract and of each fraction were concentrated through rotary evaporation at 35 °C. The resulting dry residue was then dissolved in 700 μL of 99.5% deuterated water (D_2_O) or methanol (MeOD) for NMR analyses. The ^1^H NMR and ^13^C NMR spectra were acquired using a Bruker Avance DRX-400 spectrometer (400 MHz) with a 5 mm dual ^1^H/^13^C probe head. The standard pulse sequences provided by the Bruker Software NMRlib 2.0 (Brüker, Wissembourg, France) were used. A typical ^1^H NMR spectrum consisted of 32 scans, and 2,2,3,3-tetradeuterio-3-(trimethylsilyl)-propanoic acid sodium salt served as an internal standard. The identification of major solutes present in sea-fennel extracts or fractions was determined by comparing the NMR spectra with external standards. For ^13^C (J-mod) and 2D homo- and heteronuclear NMR analyses (COSY, HMBC, HMQC, TOCSY), experiments were conducted at 298°K on a Brüker Avance III HD500 spectrometer equipped with an inverse 5 mm TCI cryoprobe (^1^H, ^13^C, ^15^N) with a z gradient. The data were processed using the TopSpin v. 4.0 program (Bruker).

### 2.12. Statistical Analyses

All extractions and assays were conducted in triplicate. The results are expressed as mean ± standard deviation (SD), and the means were compared by using a one-way analysis of variance (ANOVA) followed by Duncan’s multiple range tests performed using the ‘‘Statistica v. 5.1” software (Statsoft, 2008, Tulsa, OK, USA). The differences between individual means were deemed to be significant at *p* < 0.05. The IC_50_ values were obtained by fitting the data with a sigmoidal curve using the GraphPad Prism v. 5.0 program.

## 3. Results

### 3.1. Total Phenolic Content

**Figure 2:**
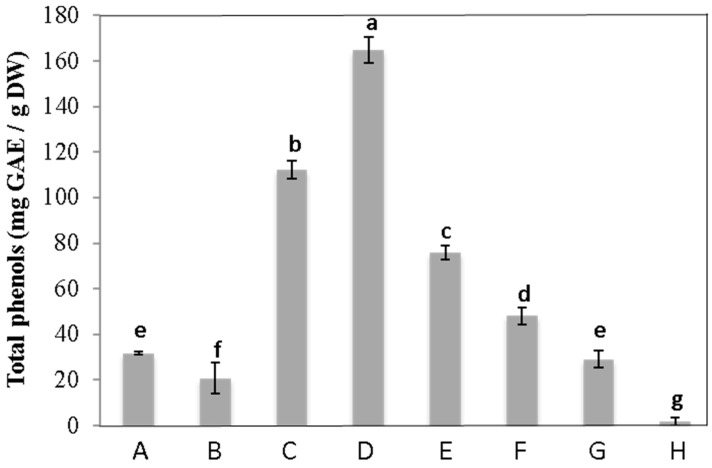
Total phenolic content (mg GAE/g DW) of *C. maritimum* crude extract and fractions. A: crude extract; B: water fraction; C: MeOH_20_ fraction; D: MeOH_40_ fraction; E: MeOH_60_ fraction; F: MeOH_80_ fraction; G: MeOH_100_ fraction; and H: Ethyl acetate fraction. Means ± standard deviations of three replicates are represented, and different letters above the bars indicate significantly different means (*p* < 0.05).

The crude extract of *C. maritimum* showed a high phenolic amount, with 33.3 mg GAE.g^−1^DW (Figure 2). After the fractionation of sea-fennel leaf extract, phenolics were detected in the three fractions eluted with 20, 40, and 60% MeOH, which exhibited 3.4, 5, and 2.3 times more phenolic compounds than crude extract, respectively.

### 3.2. Antioxidant Activities

**Figure 3:**
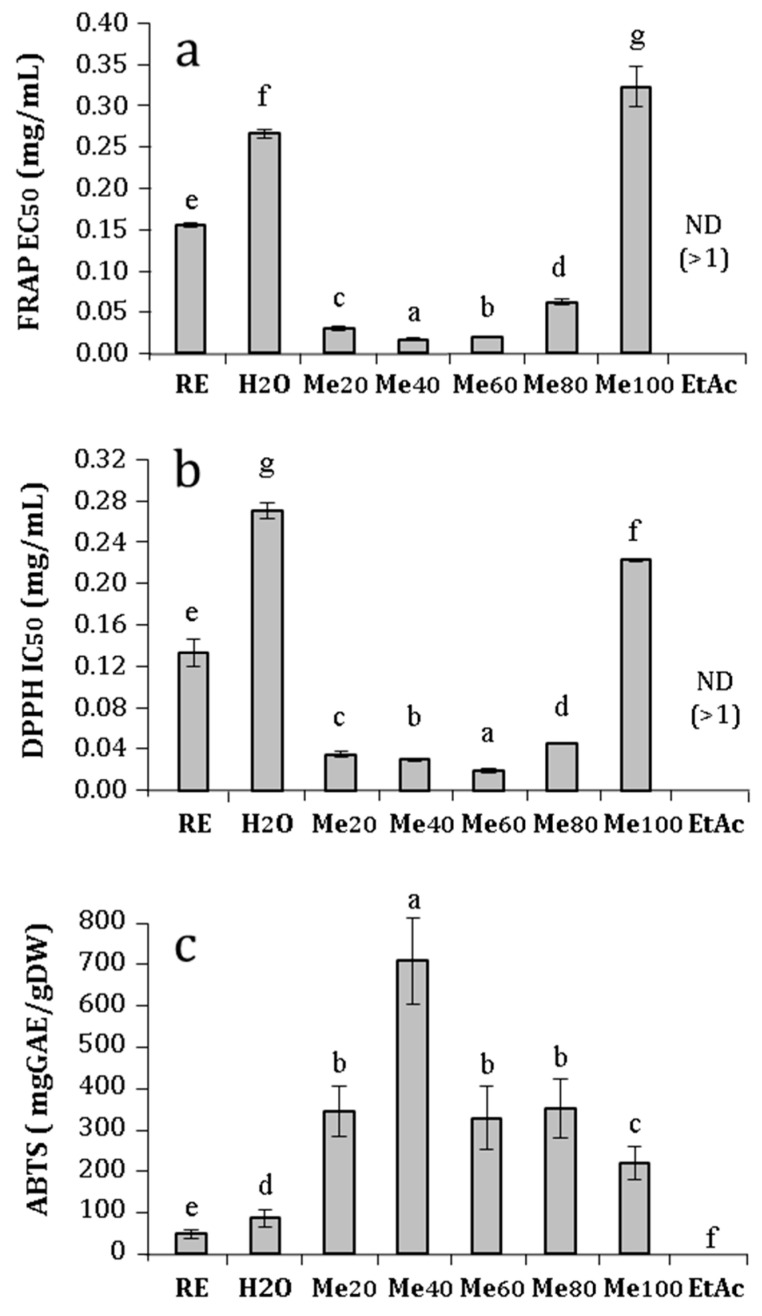
Antioxidant activities of *Crithmum maritimum* raw extract (RE) and fractions eluted with H_2_O, 20% MeOH (Me20), 40% MeOH (Me40), 60% MeOH (Me60), 80% MeOH (Me80), 100% MeOH (Me100), and ehtylacetate (EtAc). (**a**) Ferric reducing capacity (EC50 in mg/mL); (**b**) radical scavenging activity against DPPH (IC50 in mg/mL); and (**c**) radical scavenging activity against ABTS (mg GAE/g DW). Means ± standard deviations of three replicates are represented, and different letters above the bars indicate significantly different means (*p* < 0.05).

The crude extract of *C. maritimum* exhibited high antioxidant activities with EC_50_ value of 0.152 mg·mL^−1^ for FRAP bioassay (Figure 3a). After the fractionation of sea-fennel extract, MeOH_20_, MeOH_40_, MeOH_60_, and MeOH_80_ fractions exhibited a strong reducing capacity, with FRAP EC_50_ values 5.1, 7.2, 6.3, and 2.7 times lower than that of the crude extract, respectively. The same trend was obtained with the DPPH assay, where MeOH_20_, MeOH_40_, MeOH_60_, and MeOH_80_ fractions showed IC_50_ values 3.6, 4.4, 6.7, and 3 times lower than that of the crude extract (0.136 mg·mL^−1^), respectively (Figure 3b). For these two bioassays, medium polar fractions (namely MeOH_40_ and MeOH_60_ fractions) were the most active ones. ABTS radical scavenging capacity was distributed in almost all the fractions since every fraction eluted with methanol solution exhibited a strong activity (higher than 200 mg GAE·g^−1^ DW) (Figure 3c). Thus, MeOH_20_, MeOH_40_, MeOH_60_, MeOH_80_, and MeOH_100_ fractions showed 7, 10.8, 6.8, 7.1, and 4.6 times higher activities than the crude extract (51.7 mg GAE·g^−1^ DW). Conversely, the first and last fractions (namely those eluted with H_2_O and ethyl acetate) showed very low or hardly detectable activities using every antioxidant bioassay.

### 3.3. Other Biological Activities

#### 3.3.1. Anti-Ageing Activity

Sea-fennel crude extract exhibited an appreciable activity against tyrosinase, with a value of 235 mg KAE·g^−1^ DW. Of the seven fractions eluted from this extract, the last four exhibited a significantly higher activity than that of the crude extract (Table 1). Among them, the MeOH_100_ and ethyl acetate fractions appeared the most active, with more than 600 mg KAE·g^−1^ DW. Moreover, neither raw extracts nor fractions of sea-fennel aerial parts showed anti-melanogenic properties on B16 4A5 melanoma cells. 

#### 3.3.2. Anti-Inflammatory Activity

Although sea-fennel raw extract did not show any capacity to inhibit NO production by RAW 264.7 macrophages, two of its fractions were able to reduce this indicator of inflammation (Table 1). Of note, the fraction eluted with 60% MeOH showed the strongest NO inhibitory power, with an IC_50_ value (6.41 ± 0.37 μg/mL) four times lower than that of the positive control L-NAME (27.81 ± 1.93 μg/mL).

#### 3.3.3. Neuroprotective Activity

Sea-fennel extract and fractions were evaluated for their capacity to inhibit AChE. However, neither the raw extract nor its fractions exhibited any inhibitory effect on AchE.

#### 3.3.4. Antidiabetic and Anti-Obesity Activities

Pancreatic lipase plays a crucial role in the digestion and absorption of triglycerides. Inhibiting this enzyme is a widely used method to determine the potential efficacy of natural substances in averting obesity. Here, neither extracts nor fractions showed inhibitory activity on rat lipase enzyme in vitro. Then, their capacity to inhibit α-amylase and α-glucosidase was assessed. Though sea-fennel raw extract was inactive on these enzymes, two fractions strongly inhibited α-glucosidase (Table 1). Thus, MeOH_20_ and MeOH_80_ fractions exhibited a hundred-fold higher inhibition than the standard glucosidase inhibitor acarbose. However, no amylase inhibition could be detected in these fractions.

### 3.4. Solute Identification

**Figure 4:**
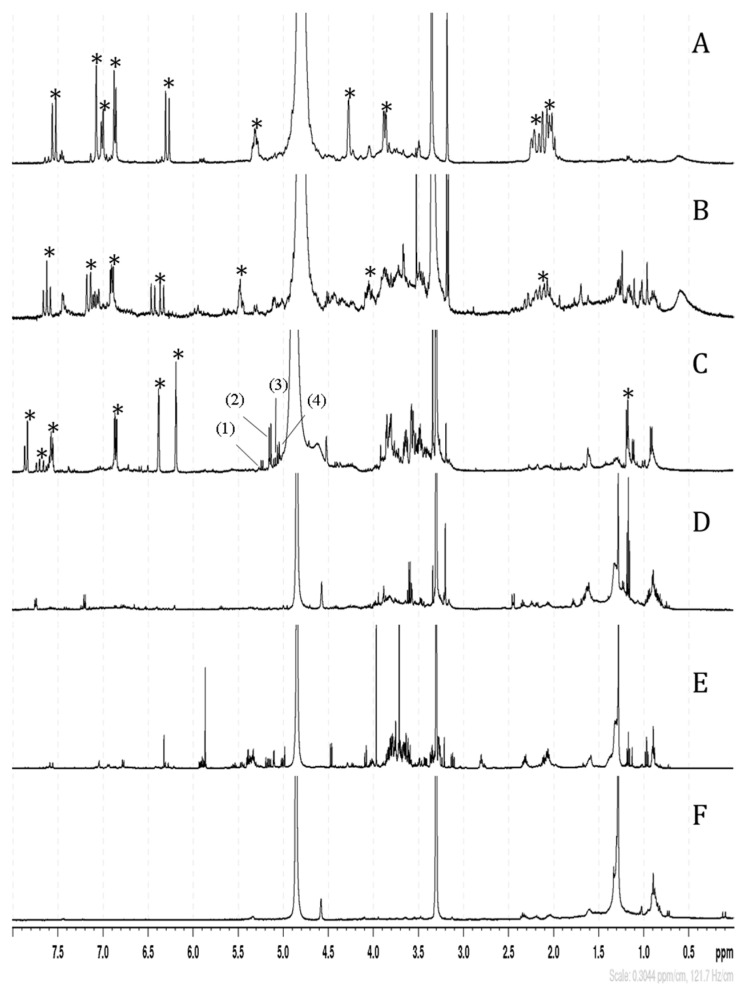
^1^H NMR spectra of *C. maritimum* bioactive fractions eluted with 20% MeOH (**A**), 40% MeOH (**B**), 60% MeOH (**C**), 80% MeOH (**D**), 100% MeOH (**E**), and ethyl acetate (**F**). Stars in spectra (**A**–**C**) indicate signals of chlorogenic acid, 3,5-dicaffeoylquinic acid, and quercetin glycosides, respectively. On the latter spectrum (**C**), specific signals assigned to quercetin 3-*O*-robinobioside (1), quercetin 3-*O*-glucoside (2), quercetin 3-*O*-galactoside (3), and quercetin 3-*O*-rutinoside (4) are indicated.

Following the purification of the extract, the active fractions were analyzed using NMR spectroscopy. The ^1^H-NMR spectrum of the MeOH_20_ fraction displayed chlorogenic acid signals predominantly (Figure 4A). In the MeOH40 fraction, characteristic signals of dicaffeoyl quinic acids, such as 3,5-di-*O*-caffeoylquinic acid, were found (Figure 4B). Signals between 6 and 7.2 ppm in the ^1^H-NMR spectrum of the MeOH_60_ fraction indicated the presence of quercetins (Figure 4C), with the two doublets between 5 and 5.2 ppm suggesting glycosylated quercetins. Subsequent HPLC purification yielded four individual compounds. Following the acid hydrolysis of these compounds, the first one provided quercetin, glucose, and rhamnose, the second provided quercetin and galactose, the third provided quercetine and glucose, and the fourth provided quercetine, glucose, and galactose. Finally, ^13^C- and 2D NMR experiments confirmed these results and led us to identify unequivocally the four glycosylated quercetins, namely quercetin 3-*O*-rutinoside, quercetin 3-*O*-galactoside, quercetin 3-*O*-glucoside, and quercetin 3-*O*-robinobioside (Supplementary Data). The MeOH_80_ fraction showed small signals in the 6–8 ppm region and more pronounced ones between 1 and 1,5 ppm on its ^1^H-NMR spectrum, corresponding to aromatic and aliphatic protons, respectively (Figure 4D). Finally, the MeOH_100_ and ethyl acetate fractions contained fewer polar compounds with aliphatic protons, as indicated by the intense signals in the 1–2 ppm region (Figure 4E,F).

## 4. Discussion

Numerous studies have evaluated the antioxidant potential of raw extracts from *C. maritimum* [16,18,21,31]. Nevertheless, there is a lack of comprehensive investigation into the molecules accountable for these properties. Three antioxidant bioassays have been used in this study to highlight different antioxidant mechanisms: ABTS and DPPH radical scavenging, as well as ferric reducing power. Additionally, for the first time in this species, biological activities of medical interest have been assessed in a raw extract and its fractions, and major compounds in these fractions have been characterized with H1-NMR to identify the possible bioactive metabolites.

The raw extract of *C. maritimum* displayed strong antioxidant activities, supporting previous findings on other sea-fennel populations [21]. Through the bioguided fractionation of this extract, the antioxidant capacity was successfully isolated into four primary fractions:

- The fraction eluted with 40% MeOH showed the most significant antioxidant activity, as revealed by ABTS-scavenging and FRAP assays. NMR analyses of this fraction indicated the prevalence of 3,5-dicaffeoylquinic acid (syn. isochlorogenic acid). Such a compound has already been reported in *C. maritimum* [31], as well as in many other halophytic species [32]. Nevertheless, the literature on its antioxidant activity is still scarce [33], compared to that of its natural isomer chlorogenic acid. Here, we suggest that the abundance of 3,5-dicaffeoylquinic acid accounts for the very strong antioxidant capacity of the MeOH_40_ fraction, as it has been reported previously in another halophytic species [34]. Of note, this fraction also inhibited NO production by LPS-stimulated RAW 264.7 cells, confirming the potent anti-inflammatory action of isochlorogenic acid [35];
- The major compounds characterized in the MeOH_60_ fraction were four glycosylated quercetins: quercetin 3-*O*-glucoside, quercetin 3-*O*-robinobioside, quercetin 3-*O*-galactoside, and quercetin 3-*O*-rutinoside. Considering the strong antioxidant power of quercetin and its derivatives [36], these four flavonols are likely responsible for the marked antioxidant activity of this fraction. Quercetin 3-*O*-glucoside is widely represented in the plant kingdom, and in particular in halophytes [37]. Jallali et al. [18] have reported its presence in sea fennel, conferring acetonic extract with a strong antioxidant activity. Moreover, Kong et al. [38] reported the anti-obesity action of this flavonol isolated from the salt-marsh plant *Salicornia herbacaea*, which was not confirmed in our work with a lipase bioassay. Conversely to quercetin 3-*O*-glucoside, the 3-*O*-rutinoside derivative of quercetin (=rutin or rutoside) has been less documented in the literature, compared to that of kaempferol or luteolin. In halophytes, the presence of rutin was reported in *Crithmum maritimum* [18,39,40], as well as in *Calystegia soldanella* [41] and *Carpobrotus edulis* [42]. Quercetin 3-*O*-galactoside has been identified previously in other (non halophytic) members of the Apiaceae family [43,44], but never in sea fennel. Moreover, quercetin 3-*O*-robinobioside has only been found in three species belonging to Fabaceae, Lamiaceae, and Rosaceae [45,46,47], and is reported here for the first time in the Apiaceae family.

Of note, this MeOH_60_ fraction exhibited a very strong NO-inhibitory activity, even more powerful than the standard anti-inflammatory L-NAME. This result is in agreement with the well-known protective effect of quercetin and its derivatives against the inflammation process [48,49], and strongly suggests that the anti-inflammatory activity of the MeOH_60_ fraction of sea-fennel extract is due to these flavonols.

- Similar analyses allowed us to elucidate the antioxidant activity of the MeOH20 fraction. The major constituent detected here was chlorogenic acid, a widely recognized antioxidant compound previously reported in C. maritimum [21]. Additionally, this fraction exhibited a remarkable anti-glucosidase activity. Chlorogenic acid has been shown to stimulate glucose uptake in skeletal muscle, thus improving glucose metabolism and preventing diabetes manifestations [50]. Our results are consistent with such observations and, owing to the richness of sea fennel in chlorogenic acid, reinforce the potential antidiabetic effect of dietary sea fennel. However, interestingly, that fraction did not exhibit any anti-lipase activity though chlorogenic acid has been reported earlier to prevent obesity manifestations [51];
- The sea-fennel extract fraction eluted with 80% methanol showed a strong antioxidant activity as well as a marked inhibition of glucosidase. The constituents of this fraction have not been completely elucidated, but its NMR spectrum suggests the presence of less polar phenolics and compounds with short aliphatic chains.

The anti-tyrosinase activity was determined here for the first time in sea fennel by assessing the inhibition of L-DOPA oxidation to dopaquinone (diphenolase activity). Interestingly, the moderate activity of crude extract was concentrated in four fractions, indicating the contribution of several constituents of differing structures in that activity. Thus, the four least polar fractions exhibited the most powerful anti-tyrosinase activity. Moreover, the inhibition level was quite close to that of the kojic acid standard, and much higher than that recently reported in other halophytic species [5]. Since tyrosinase catalyzes oxidative reactions, the inhibitory activity of MeOH_60_ and MeOH_80_ fractions is likely due to their major antioxidant phenolic compounds, as reported by Chang [52]. For example, one of the quercetine glycosides identified in the MeOH60 fraction, namely quercetine 3-*O*-galactoside, was reported to inhibit tyrosinase [53]. Additionally, of note was the highest anti-tyrosinase activity exhibited by the non-antioxidant fractions eluted with 100% MeOH and ethyl acetate. Their composition has not been completely elucidated yet, but preliminar NMR analyses suggest that molecules involved in anti-tyrosinase activity here are likely acyclic compounds with carbon chains such as terpenoids. Therefore, our study confirms in part that a positive correlation could be made between antioxidant and anti-tyrosinase activities, as reported previously by Choi et al. [54] and Lee et al. [55]. However, a contribution by other (non-antioxidant) substances to tyrosinase inhibition should not be excluded.

The well-known neurotransmitter acetylcholine facilitates the recovery of neuronal function after brain injury. Therefore, it is convenient to investigate the powerful neuroprotective effect of natural compounds by inhibiting AChE activity [56]. Here, sea-fennel extract and fractions did not exhibit any capacity to inhibit AChE. This result was consistent with a previous work, reporting that such activity could only be found in essential oils of Apiaceae flowers [57,58].

## 5. Conclusions

On the whole, we demonstrated that the antioxidant activity of *C. maritimum* hydroalcoholic extract is present in its mid-polar fractions, with chlorogenic acid, 3,5 dicaffeoyl quinic acid, and quercetin glycosides being the major contributors. Of these fractions, some also exhibited antidiabetic or anti-inflammatory properties. On the other hand, the less polar fractions are the most active against tyrosinase, and additional investigations are being conducted to ascertain the compounds (presumably terpenoids) accountable for this activity. Additionally, no anti-melanogenic nor anti-obesity properties could be found in sea-fennel hydromethanolic extract and its fractions. Overall, the findings from this study should underscore the nutraceutical value of *C. maritimum* extract or antioxidant secondary metabolites as potent agents against inflammatory, diabetes-related, and ageing processes.

## Figures and Tables

**Table 1:** Anti-tyrosinase (diphenolase inhibition), anti-inflammatory (inhibition of NO production in RAW 264.7 macrophages), and antidiabetic (alpha-glucosidase inhibition) activities of sea-fennel polar extract and its fractions. Means ± SDs of three replicates are presented, and different letters indicate significantly different means (*p* < 0.05). ND, not detected.

	Anti-Tyrosinase (mgKAE/gDW)	NO Inhibition (IC_50_, μg/mL)	Anti-α Glucosidase (IC_50_, mg/mL)
Raw extract	234.29 ± 48.56 c	ND	ND
MeOH20	151.12 ± 74.89 c	ND	0.02 ± 0.01 b
MeOH40	226.37 ± 91.23 c	89.54 ± 2.16 a	ND
MeOH60	531.46 ± 68.42 b	6.41 ± 0.37 c	ND
MeOH80	563.14 ± 26.94 b	ND	0.04 ± 0.00 b
MeOH100	637.02 ± 29.23 a	ND	ND
EtAc	626.19 ± 41.13 a	ND	ND
L-NAME		27.81 ± 1.93 b	
Acarbose			3.14 ± 0.09 a

## Data Availability

The original contributions presented in the study are included in the article/Supplementary Materials, further inquiries can be directed to the corresponding author.

## Supplementary Materials

The following supporting information can be downloaded at https://www.mdpi.com/article/10.3390/foods13091294/s1. Supplementary Data: 1. NMR data (500 MHz, CDCl_3_) of 3,5-dicaffeoylquinic acid isolated in the MeOH_40_ fraction. 2. NMR data (500 MHz, CDCl_3_) of the three quercetine derivatives isolated in the MeOH_60_ fraction.

## Abbreviation

AChE, acetylcholinesterase; ATChI, acetylthiocholine iodide; BHT, Butylated hydroxytoluene; DMSO, Dimethyl sulphoxide; DPPH, 1,1-diphenyl-2-picrylhydrazyl; DTNB, (5,5′-dithiobis-(2-nitrobenzoic acid); EtAc, ethyl acetate; FBS, fetal bovine serum; L-NAME, N(gamma)-nitro-L-arginine methyl ester; L-DOPA, L-3,4 dihydroxyphenylalanine; LPS, lipopolysaccharide; MeOH, methanol; PDA, photodiode array; RPMI, TPTZ, 2,4,6-Tris(2-pyridyl)-s-triazine.
