# Supplementary material for: A Characterization of Biological Activities and Bioactive Phenolics from the Non-Volatile Fraction of the Edible and Medicinal Halophyte Sea Fennel (Crithmum maritimum L.)
Source: Foods. 2024 Apr 23;13(9):1294. doi: 10.3390/foods13091294 (PMC11083217; doi:10.3390/foods13091294)
Supplement: Supplementary file 1 [file foods-13-01294-s001.zip › foods-2911059-supplementary.pdf]

Supplementary Data

1. NMR spectroscopic data (500 MHz, $CDCl_3$) for 3,5-di-*O*-caffeoylquinic acid identified in $MeOH_{40}$ fraction of sea fennel extract.
(d: doublet, dd: doublet of doublets, m: multiplet, brs: broad singlet).

**Compound**	**3,5-di-*O*-caffeoylquinic acid**	
Position	δ_H (J in Hz)	δ_C, Type
1	-	74.7, qC
2	2.35/2.13 (m)	36.0, CH_2
3	5.45/5,36 (m)	72.5, CH
4	3.96 (dd, 9.9)	70.5, CH
5	5.36/5.45 (m)	72.1, CH
6	2.13/2.35 (m)	38.6, CH_2
7	7.60 (d, 15.9)	177.3, COOH
1′/1′′	-	127.9/127.8, qC
2′/2′′	7.06 (brs)	115.1, CH
3′/3′′	-	146.8, qC
4′/4′′	-	149.6/149.5, qC
5′/5′′	6.77 (dd, 1.2)	116.5, CH
6′/6′′	6.97/6.96 (dd, 2.0)	123.1/123.0, CH
7′/7′′	7.60/7.57 (d, 15.9)	147.3/147.0, CH
8′/8′′	6.35/6.26 (d, 15.9)	115.5/115.2, CH
9′/9′′		168.9/168.3, qC

2. NMR spectroscopic data (500 MHz, $CDCl_3$) for 2 glycosylated quercetines identified in $MeOH_{60}$ fraction of sea fennel extract. 1, Quercetin-3-*O*-rutinoside; 2, Quercetin-3-*O*-glucoside.
(d: doublet, dd: doublet of doublets, m: multiplet).

**Compounds**	**1**		**2**	
Position	δ_H (J in Hz)	δ_C, Type	δ_H (J in Hz)	δ_C, Type
2	-	159.3, qC	-	159.3, qC
3	-	135.6, qC	-	135.2, qC
4	-	179.4, qC	-	179.4, qC
5	-	162.9, qC	-	163.1, qC
6	6.19 (d, 1.9)	100.1, CH	6.19 (d, 1.9)	100.2, CH
7	-	166.6, qC	-	167.0, qC
8	6.38 (d, 1.9)	94.9, CH	6.38 (d, 1.9)	94.9, CH
9	-	158.6, qC	-	158.5, qC
10	-	105.5, qC	-	105.1, qC
1′	-	123.1, qC	-	123.2, qC
2′	7.66 (d, 8.6)	117.6, CH	7.66 (d, 8.6)	117.3, CH
3′	-	145.9, qC	-	145.0, qC
4′	-	149.9, qC	-	149.0, qC
5′	6.86 (d, 8.6)	116.1, CH	6.86 (d, 8.6)	115.2, CH
6′	7.62 (dd, 1.9)	123.5, CH	7.57 (dd, 1.9)	123.1, CH
1″	5.10 (d, 7.9)	104.8, CH	5.12 (d, 7.9)	104.4, CH
2″	3.27 (dd, 9.25)	75.7, CH	3.47 (dd, 9.25)	75.5, CH
3″	3.40-3.46 (m)	78.2, CH	3.39 (m)	77.9, CH
4″	3.40-3.46 (m)	71.4, CH	3.42 (m)	71.2, CH
5″	3.38 (m)	77.2, CH	3.38 (m)	77.2, CH
6″	3.71 (m)-3.90 (m)	68.6, CH_2	3.72 (m)-3.87 (m)	68.6, CH_2
1‴	4.50 (d, 1.9)	102.4, CH		
2‴	3.60 (dd,)	72.2, CH		
3‴	3.51 (m)	72.1, CH		
4‴	3.25 (m)	73.9, CH		
5‴	3.42 (m)	69.7, CH		
6‴	1.10 (d, 5.9)	17.9, CH		
